# Reporting ethical approval in health and social science articles: an audit of adherence to GDPR and national legislation

**DOI:** 10.1186/s12910-021-00664-w

**Published:** 2021-07-15

**Authors:** Kjell Asplund, Kerstin Hulter Åsberg

**Affiliations:** 1 Department of Public Health and Clinical Medicine, Umea University, Reimersholmsgatan 59, 117 40 Stockholm, Sweden; 2 Department of Neuroscience, Uppsala University, Uppsala, Sweden

**Keywords:** Research ethics, Ethics approval, GDPR, Health sciences, Social sciences

## Abstract

**Background:**

Previous studies have indicated that failure to report ethical approval is common in health science articles. In social sciences, the occurrence is unknown. The Swedish Ethics Review Act requests that sensitive personal data, in accordance with the EU General Data Protection Regulation (GDPR), should undergo independent ethical review, irrespective of academic discipline. We have explored the adherence to this regulation.

**Methods:**

Using the Web of Science databases, we reviewed 600 consecutive articles from three domains (health sciences with and without somatic focus and social sciences) based on identifiable personal data published in 2020.

**Results:**

Information on ethical review was lacking in 12 of 200 health science articles with somatic focus (6%), 21 of 200 health science articles with non-somatic focus (11%), and in 54 of 200 social science articles (27%; *p* < 0.001 vs. both groups of health science articles). Failure to report on ethical approval was more common in (a) observational than in interventional studies (*p* < 0.01), (b) articles with only 1–2 authors (*p* < 0.001) and (c) health science articles from universities without a medical school (*p* < 0.001). There was no significant association between journal impact factor and failure to report ethical approval.

**Conclusions:**

We conclude that reporting of research ethics approval is reasonably good, but not strict, in health science articles. Failure to report ethical approval is about three times more frequent in social sciences compared to health sciences. Improved adherence seems needed particularly in observational studies, in articles with few authors and in social science research.

**Supplementary Information:**

The online version contains supplementary material available at 10.1186/s12910-021-00664-w.

## Background

Failure to report on informed consent and approval by an ethics review board has been described to be frequent in clinical research, even in prestigious journals [1]. Recent assessments have shown marked variations between research areas in the proportion of articles lacking information on external ethics review. The proportion has been reported to range from 6 per cent in nursing research [2] to 48 per cent in pediatric surgery [3] and 50 per cent in otolaryngology [4]. Previous studies have also shown a considerable between-journal variation in the proportion of clinical articles reporting on ethical approval [1], even within one and the same discipline [5].

How research ethical review is regulated varies considerably between countries. Whereas many countries have legislation or other nationwide regulation on research ethics review, ethics review committees/institutional review boards are local or regional in most countries. Numerous studies based on multinational research projects describe widely different outcomes between countries when one and the same application is assessed; this has been called “ethics review roulette” [6].

In the European Union, the General Data Protection Regulation (GDPR) [7] covers all treatments of personal data in EU and provides common definitions of what are personal data; it is supposed to be complemented by national laws. As to research ethics, GDPR is implemented in the Swedish Ethical Review Act [8]. The Act also specifies a centralized system for ethical review with a national Ethical Review Authority [9]. According to the Act, ethical review is mandatory for (a) research that involves personal data that in the Swedish Act are termed “sensitive”, identical to data defined in GDPR, article 9.1, as “special categories of personal data” [10, 11], (b) research that involves physical encroachment on an individual or uses a method that aims to affect the subject physically or psychologically, and (c) studies on biological material traceable to specific individuals. In the Act, it is also stated that it applies to personal data on crimes, convictions and certain other specified legal decisions. There may be pros and cons of strict regulation of research ethics reviews, including law-making, and the Act and its limitations has been debated in the law literature (e.g. [12, 13]).

The present study contributes with empirical information on adherence to GDPR and the Swedish Act as to reporting of approval by the Ethics Review Authority (or previous Regional Review Boards) in published scientific articles. We have compared failures to report this information in health and social science articles and explored in what settings failures may be particularly frequent.

## Methods

To identify relevant articles, we screened Science Citation Index Expanded and Social Sciences Citation Index in the Web of Science databases [14], using “Sweden” in the address field. Consecutive articles published from January 1st 2020 onwards were screened. Articles were included if they fulfilled the following criteria: (a) health or social sciences, (b) study with original data, (c) containing personal data according to GDPR [7]: racial or ethnic origin, political opinions, religious or philosophical beliefs, trade union membership, genetic data, biometric data for the purpose of uniquely identifying a natural person, data concerning health or data on sex life or sexual orientation, (d) personal data on crimes, convictions and certain other legal decisions as specified in the Ethical Review Act, and (e) personal data collected in Sweden; this latter criterion was used because, in multinational collaborations, information was usually not provided about in which country the analyses had been performed. Each article was assessed by two reviewers; discrepancies were solved by consensus discussion.

Exclusion criteria were: (a) articles with completely anonymized individual data, (b) articles not listed in GDPR as one of the “special categories of personal data” [7], for instance studies of attitudes, experiences and working conditions of healthcare and social services staff without personal data on the staff’s health, and (c) articles based on information on deceased persons. It should be noted that our study did not include jurisprudence or humanities research.

In each of three categories, the first 200 consecutive articles in the Web of Science databases were included: health science with somatic focus, health science with non-somatic focus, and social sciences. Thus, a total of 600 articles with personal data were included. If an article was not available online at the university library (n = 21; 3%), it was replaced by the next consecutive article available.

The articles were reviewed for information on approval by the Swedish Ethical Review Authority or its predecessors Regional Ethical Review Boards, including registration number. In articles that did not report an ethical approval but referred to a previous publication from the same research project, the latter was reviewed for possible reporting of ethical approval. In addition, we abstracted information on publication journal, study design, research discipline, number of authors, and the corresponding author’s institution. Impact factors of the journals were retrieved from Journal Citation Reports database [15].

## Results

**Fig. 1:**
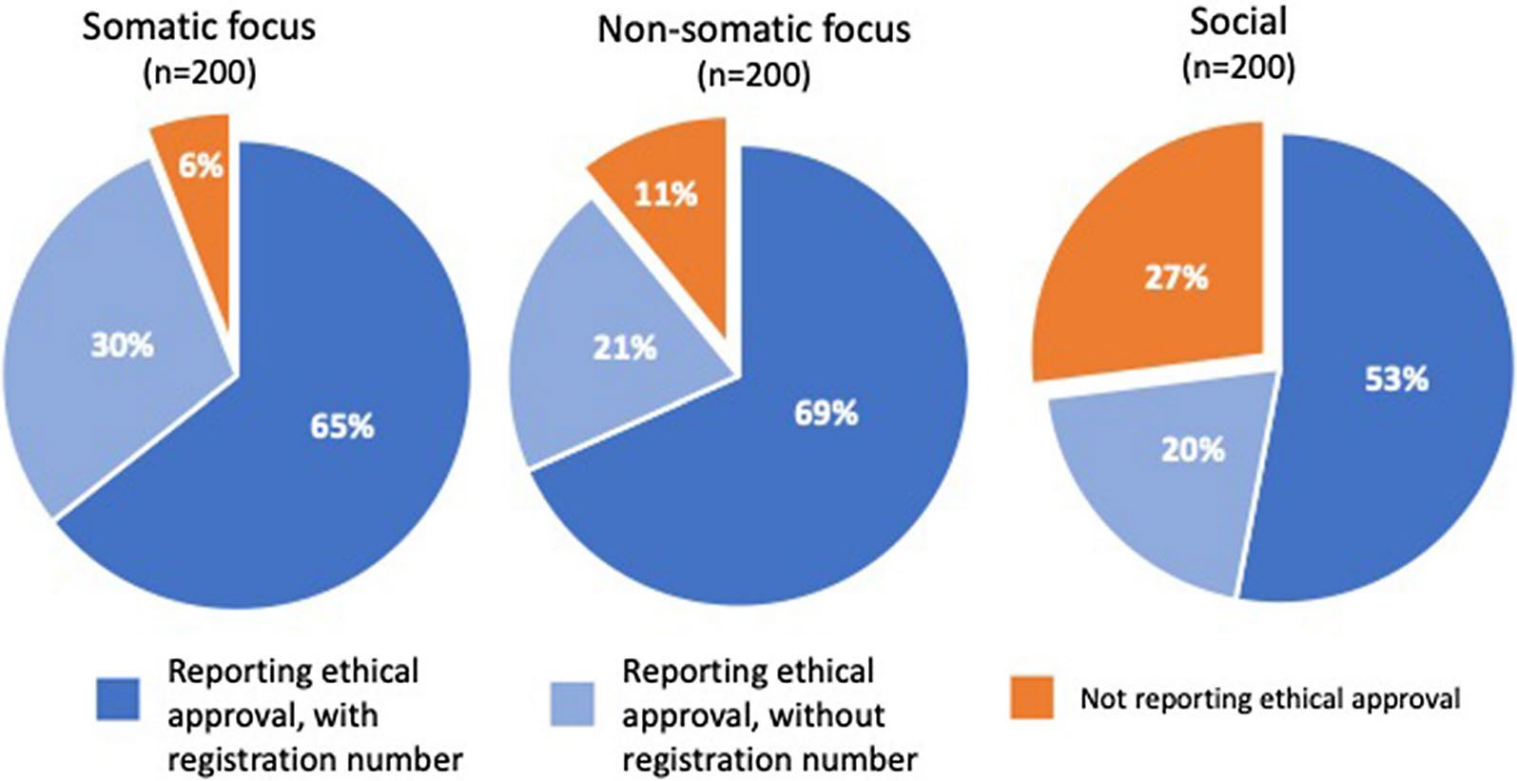
Proportion of articles reporting on ethical approval (with and without registration number) in health sciences with somatic and non-somatic focus and social sciences articles

All articles fulfilling the inclusion criteria were published either in English or Swedish. Of the 600 articles (from 361 different journals), 87 (14.5 per cent) lacked information on approval by the Ethical Review Authority or a Regional Ethical Review Board. As shown in Fig. 1 and Table 1, the proportion without information on ethical approval was modestly higher in health studies with a non-somatic focus compared to those with a somatic focus (11% vs. 6%, statistically not significant). It was about three times higher in social science articles (27%; chi-square test *p* < 0.001 vs. both groups of health science articles). Of articles reporting on ethical approval, about three quarters also reported registration number of the approval, making it traceable (Fig. 1).

**Table 1:** Lack of information on ethical approval in health and social sciences articles with Swedish research persons

Study characteristics	Health sciences, somatic focus (n = 200)		Health sciences, non-somatic focus (n = 200)			Social sciences (n = 200)		Total (n = 600)	
	Information missing	Fisher’s exact test, *p*-value	Information missing	Fisher’s exact test,*p*-value		Information missing	Fisher’s exact test, *p*-value	Information missing	Fisher’s exact test, *p*-value
All articles	12/200; 6%	–	21/200; 11%	–		54/200; 27%	–	87/600; 15%	–
*Study type*									
Intervention	2/77; 3%	Non-sign	2/20; 10%	Non-sign		3/24; 13%	Non-sign	7/121; 5%	< 0.01
Observation	10/123; 8%		19/180; 11%			51/176; 29%		80/479; 17%	
*No. of authors*									
1–2	0/5; 0%	a	6/16; 38%	< 0.01		24/57: 43%	< 0.01	30/78: 38%	< 0.001
≥ 3	12/195; 6%		15/184: 8%			30/143; 20%		57/522; 11%	
*University*									
With medical school^b^	12/194; 6%	a	12/165; 7%	< 0.01		28/116; 27%	Non-sign	54/475; 11%	< 0.001
Other^c^	0/6; 0%		9/35; 26%			26/84; 31%		35/125; 28%	

Non-sign. *p* > 0.05

^a^Not possible to calculate because of low numbers

^b^Including university hospitals

^c^Including 7 articles from non-academic institutions

In health science articles with somatic focus, the proportion lacking information on ethical approval was low in all subcategories: surgical focus (3 of 39; 8%), non-surgical focus (9 of 138; 7%), and laboratory focus (0 of 13). The category health science articles with non-somatic focus was heterogenous. The proportion of articles without information on ethical approval did not differ significantly between the subgroups of mental and neurological disorders (9 of 84; 11%), nursing research (2 of 31; 6%) and the collective group of other disciplines (10 of 85; 12%). As shown in Table 2, in the social science category, significant deviations from the mean (27%) were observed for research on the elderly (0%; significantly lower) and the heterogenous group “Other” (61%; significantly higher) which included a wide range of disciplines such as political science, economics, linguistics, sports research, communication, environmental and transportation research. Low statistical power hampered many of the comparisons of subcategories.

**Table 2:** Lack of information on ethical approval in social sciences articles by disciplines

Discipline	Social sciences (n = 200)	
	Information missing	Odds ratio (95% conf. intervals)
All	54/200 (27%)	1.00
Sociology and related^a^	26/93; 31%	1.05 (0.61;1.82)
Psychology and related	10/32; 31%	1.23 (0.55–2.76)
Alcohol and substance abuse	1/12; 8%	0.25 (0.03–1.95)
Education	6/20; 30%	1.16 (0.42–3.17)
Elderly	0/25; 0%	0.11 (0.01–0.82)^c^
Other^b^	11/18; 61%	4.25 (1.57–11.5)

Odds ratios for information on ethical approval missing, with 95% confidence intervals

^a^Including social work, criminology, work environment

^b^Including political science, economics, linguistics, sports research, communication, environmental research

^c^0 instances of missing information replaced by 1 to calculate odds ratio

We classified the articles as observational and interventional studies, respectively. Observational studies included cross-sectional, case–control and cohort studies, as well as studies using qualitative methods and participant observations. Information on ethical approval was lacking about three times more often in observational compared to interventional studies (17% vs. 5%; *p* < 0.01) (Table 1). Most interventional studies were in health research with a somatic focus, and the statistically significant overall difference was mainly driven by the difference in this category. Further subgrouping by study design is shown in Additional file 1. Many of the subgroups were too small to permit statistical analyses of differences between groups. Therefore, statistical comparisons were performed for the three most common study designs only. Failure to report ethical approval was significantly more common in social science than in health science articles with cross-sectional design (25% vs. 9%; *p* < 0.001 by Fisher’s exact test) and cohort design (33% vs. 9%; *p* < 0.001), The same tendency was observed for qualitative studies (25% vs. 10%), although the difference was not statistically significant (*p* = 0.10).

When an article had only 1 or 2 authors, lack of information on ethical approval was nearly four times as common as when there were 3 or more authors; the difference was highly statistically significant (*p* < 0.001; Table 1).

The affiliation of the corresponding author or, in multinational studies, the author who was mainly responsible for the Swedish component of the study, was dichotomized into universities with and without a medical school (education of physicians). The great majority of studies were from universities with a medical school (475 of 600; 79%). Overall, lack of information on ethics approval was more than twice as frequent in universities without a medical school as in those with a medical school (*p* < 0.001; Table 1). The statistical significance was entirely driven by a marked difference among health science studies with a non-somatic focus.

We also explored if lack of information on ethical approval was associated with a low impact factor of the journals in which the articles had been published. The mean impact factor was highest for health science articles with somatic focus and lowest for social sciences (Table 3). Within all the three major categories, the journal impact factor was similarly distributed in articles with and without information on ethical approval.

**Table 3:** Journal impact factor for articles with and without ethical approval by research area. Means and 95% confidence intervals

Research area	Ethical approval, mean (95% CI)	
	Reported	Not reported
Health sciences, somatic focus	4.55 (4.14;4.96)	6.35 (4.05;8.65)
Health sciences, non-somatic focus	3.15 (2.71;3.59)	3.04 (2.32;3.76)
Social sciences	2.20 (2.03;2.37)	1.86 (1.60;2.12)

In 20 of the 87 articles lacking information on ethical approval, there was a commentary on research ethics or an explanation why approval by the Ethical Review Authority or the previous Regional Ethical Review Boards had not been obtained (Table 4). In 6 articles, the authors incorrectly stated that ethics approval was not required or not relevant. In 5 articles, the authors referred to approval by a local ethics committee at the institution or to local ethical guidelines (not compliant with the Swedish Ethics Review Act). In 7 articles without information on ethical approval, there were general reassurances that ethical guidelines had been adhered to and/or referral to the Helsinki Declaration. In 2 articles, it was stated that the article was based on students’ examination works, exempted from compulsory ethics review by law.

**Table 4:** Ethical comments in articles not reporting on ethical approval by the national Ethics Review Authority (or its predecessors Regional Review Boards)

Ethical comment	Health science with somatic focus	Health science with non-somatic focus	Social sciences	Total
Stating “not required” or “not relevant”	0	2	4	6
Referral to approval by local committee or to local guidelines	0	0	5^a^	5
General assurance of adherence to ethical guidelines and/or referral to the Helsinki Declaration	1	3	3	7
Student work^b^	0	2	0	2
None	11	14	42	67

^a^Including 1 study that referred to approval by an agency other than the Ethical Review Authority

^b^Student works are exempted in the Swedish Act om research ethics review

## Discussion

Our results show that information on ethics approval of research with identifiable personal data is not reported in about one in ten articles in health sciences and in more than a quarter of articles in social sciences. The risk for non-reporting is higher in observational than in interventional studies, in articles with only one or two authors than in multi-authored articles and, as to health science articles with a non-somatic focus, in universities without a medical school compared to those with a medical school. There was no apparent association with the impact factor of the journals in which the articles were published.

The Swedish Ethical Review Act applies not only to health sciences but also to social sciences, similarly to the regulation in some other countries, for instance Norway [16] and the United States [17]. Whereas lack of information on ethical approval has been reported in a limited number of previous health research studies (see Introduction), we have not been able to identify any corresponding study of ethical approval in social sciences.

The Swedish system is centralized with an Act covering all human research and a national Ethics Review Authority. Direct comparisons with previous studies on failure to report ethical approval are complicated by differences in methodology and the fact that previous studies have been limited to a few selected international journals, usually in a specific discipline. We have included consecutive studies in the Web of Science databases, irrespective of discipline but our study was restricted to research performed in one country. Our results are similar to those reported in nursing research articles (6% failures to report ethical approval; [2]) but much lower than in pediatric surgery and otolaryngology (48–50%; [3, 4] and in five prestigious medical journals in the mid-2000s (31%; [1]). Although, in many countries, more attention is being paid to research ethics issues today, there is little data to support a secular trend in the adherence to recommendations and regulations. Nonetheless, a tentative conclusion from our results is that the frequency of failure to report ethical approval in health sciences in Sweden (one in ten) is considerably lower than what has been observed in most previous studies. In social sciences, there are no data from other countries to compare with.

In the literature, there is scanty information on high-risk settings for not reporting on ethical approval. We observed that failure to report on ethical approval was much more common in articles based on observational studies than on intervention studies. This is in line with previous observations that the frequency of non-reporting is low in articles based on randomized controlled trials [1, 18] and lower in prospective than in retrospective studies [19].

In view of the previously reported between-journal variation in the proportion of articles not reporting on ethics review [1, 5], we hypothesized that failure to report was more frequent in articles published in low-impact than in high-impact journals. Our results did not, however, support this hypothesis. Within each of the three research domains, the impact factor distribution was similar whether or not ethical approval had been reported. There seems to be an opportunity also for editors of many high- and medium-impact journals to improve authors’ reporting of ethical approval when articles are based on identifiable personal data.

Articles with only one or two authors were associated with a high risk of not reporting on ethical approval. The chance of someone in the research group being experienced in ethics regulations would be greater in a larger team. In health science articles with a non-somatic focus, failure to report on ethical approval was much more frequent when the articles came from universities without a medical school, another indicator of the research environment being significant for adherence to ethics regulations. Medical research has a long tradition of ethical reviewing. The Ethics Review Act has been in place in Sweden since 2004 and, yet, it seems that adherence to the law is still far from strict in many social sciences (although it should be noted that in articles reporting social research on the elderly we did not find a single instance of failure to report ethical approval). Non-reporting seems to be particularly common in those social science disciplines where studies based on personal data are infrequent (in our analysis assembled in the “Others” subgroup). A reason for the more frequent non-reporting of ethical approval in social sciences may be less training of junior researchers in research ethics, resulting in insufficient knowledge about the legislation. Other tentative explanations for the differences between medical and social sciences are that scientific journals in medicine tend to require reporting of ethical approval more strictly; this may also apply to funders of medical research. In the international literature, the need for ethical review of social sciences studies and its legitimacy have been questioned and the procedures for ethical review of social science projects have been criticized [20, 21]; it may be speculated if limited legitimacy among the researchers is reflected in reduced adherence to the Ethical Review Act.

There are several reasons why ethical approval is not reported. Ethical approval may have been obtained but not reported in the publication. The authors may have misinterpreted the law, for instance by incorrectly assuming that it does not apply to their research. As shown by the comments in a few of the articles, the authors may wrongly think that approval by a local committee may substitute approval by the national Ethics Review Authority. The Act explicitly exempts student works, but this reason was given in only 2 of 87 articles lacking information on ethical approval. In all likelihood, the great majority of non-reporting is ascribed to lack of obtaining statutory ethical approval for research based on sensitive personal data. Our study was not designed to explore to what extent the researchers deviate from the ethical approvals that had been granted (sometimes conditional).

A limitation of our study is that there may be some classification bias. Many of the studies were multidisciplinary and we classified them by their main focus; there was therefore an element of subjectivity. Any possible classification bias could result in either exaggerated or reduced differences between research areas.

The Ethical Review Act covers not only data collected in the country but also data from other countries analyzed in Sweden. However, in multinational collaborations, exact information is usually lacking on where the analyses were performed. A limitation is therefore that some multinational collaborative studies where data have been analyzed in Sweden (with no Swedish sensitive personal data) have not been covered.

In large subgroups, the numbers were sufficient to make comparisons with appropriate statistical power; however, the numbers were small in many subgroups, resulting in low statistical power. It should also be noted that our investigation covers only one aspect of the ethical review process, concerning research participants’ integrity. Other considerations, such as those on possible risk and benefits of the research, would have required access to full documentation of each study.

A further putative limitation, pertaining particularly to social science studies, is that we included only studies with original data published in journals included in the Web of Science databases. Thus, monographs, book chapters, conference proceedings and similar literature were not included. Given that the peer review process may be less rigorous in some of this literature, we see no reason to believe that failures to report ethical approval would be considerably lower, had such literature been included in our study.

In a recent update of the Swedish Ethics Review Act, a centralized inspection function was introduced [8]. Our results may be helpful to identify research that is at high risk for being conducted without ethical approval: observational studies, particularly in social sciences, performed by only one or two authors and, in health sciences, executed in a university without a medical school.

## Conclusions

In Sweden, reporting of research ethics approval is reasonably good, but not strict, in health science articles. Failure to report ethical approval is about three times more frequent in social sciences compared to health sciences and more frequent in observational than in interventional studies, in articles with one or two authors compared with multiauthored articles and, in health science articles with a non-somatic focus, in universities without a medical school compared to those with a medical school. Contrary to our expectations, there seems to be no apparent association with the impact factor of the journals in which the articles are published. Our results may be helpful to identify research based on sensitive personal data that is at high risk for being conducted without mandatory ethical approval.

## Supplementary Information

**Additional file 1**. Lack of information on ethical approval in health and social sciences articles with Swedish research persons in studies of different designs..

## Data Availability

Primary data are available at http://www.kjellasplund.se/data-for-articles/.
