# Supplementary material for: Reporting ethical approval in health and social science articles: an audit of adherence to GDPR and national legislation
Source: BMC Med Ethics. 2021 Jul 15;22:92. doi: 10.1186/s12910-021-00664-w (PMC8284007; doi:10.1186/s12910-021-00664-w)
Supplement: Supplementary file 1 — Additional file 1. Lack of information on ethical approval in health and social sciences articles with Swedish research persons in studies of different designs. [file 12910_2021_664_MOESM1_ESM.docx]

**Additional file 1.** Lack of information on ethical approval in health and social sciences articles with Swedish research persons in studies of different designs.

| Study design | Numbers and proportions of studies lacking information  on ethical approval | | |
| --- | --- | --- | --- |
|  | Health sciences,  somatic focus | Health sciences,  non-somatic focus | Social sciences |
| *Interventional* |  |  |  |
| randomized controlled trial | 0/9; 0% | 1/3; 33% | 1/3; 33% |
| non-randomized controlled trial | 0/12; 0% | 0/3: 0% | 0/4; 0% |
| short-term experiment | 0/16; 0% | 1/10; 10% | 1/7; 14% |
| other trial design | 2/40; 5% | 0/4; 0% | 1/10; 10% |
| *Observational* |  |  |  |
| qualitative | 0/0; 0% | 4/40; 10% | 12/48; 25% |
| retrospective, incl. case-control | 1/10; 10% | 1/3; 33% | 0/3; 0% |
| cross-sectional | 4/57; 7% | 6/59; 10% | 22/79; 28% |
| cohort | 5/56; 9% | 6/74; 9% | 13/39; 33 % |
| ethnographic/participating observation | 0/0; 0% | 2/4; 50% | 4/7; 57% |
